# Adverse Childhood Experiences Predict Early Initiation of Opioid Use Behaviors

**DOI:** 10.3389/fsoc.2021.620395

**Published:** 2021-05-13

**Authors:** Honoria Guarino, Pedro Mateu-Gelabert, Kelly Quinn, Skultip Sirikantraporn, Kelly V. Ruggles, Cassandra Syckes, Elizabeth Goodbody, Lauren Jessell, Samuel R. Friedman

**Affiliations:** ^1^Institute for Implementation Science in Population Health, City University of New York (CUNY) Graduate School of Public Health and Health Policy, New York, NY, United States; ^2^Department of Population Health, New York University (NYU) Grossman School of Medicine, New York, NY, United States; ^3^Fulbright University Vietnam, Ho Chi Minh City, Vietnam; ^4^Division of Translational Medicine, Department of Medicine, New York University (NYU) Grossman School of Medicine, New York, NY, United States; ^5^U. S. Sentencing Commission, Washington, DC, United States

**Keywords:** adverse childhood experiences, childhood trauma, opioid misuse, non-medical prescription opioid use, drug use initiation, young adults (18–29 years)

## Abstract

**Introduction:** Although a substantial body of research documents a relationship between traumatic stress in childhood and the initiation of substance use later in the life course, only limited research has examined potential linkages between adverse childhood experiences (ACEs) and the initiation of non-medical prescription opioid use and other opioid use behaviors. The present study contributes to this growing body of work by investigating the association of childhood trauma with early initiation of a series of opioid use behaviors.

**Methods:** New York City young adults (*n* = 539) ages 18–29 who reported non-medical use of prescription opioids or heroin use in the past 30 days were recruited using Respondent-Driven Sampling in 2014–16. Ten ACEs were assessed via self-report with the ACE Questionnaire. Associations between number of ACEs and self-reported ages of initiating seven opioid use behaviors (e.g., non-medical prescription opioid use, heroin use, heroin injection) were estimated with multivariable logistic regression.

**Results:** Eighty nine percent of participants reported at least one ACE, and 46% reported four or more ACEs, a well-supported threshold indicating elevated risk for negative health consequences. Every increase of one trauma was associated with a 12–23% increase in odds of early initiation across the seven opioid use behaviors. Findings also document that the mean age at initiation increased with increasing risk severity across the behaviors, contributing to evidence of a trajectory from opioid pill misuse to opioid injection.

**Discussion:** Increasing number of childhood traumas was associated with increased odds of earlier initiation of multiple opioid misuse behaviors. In light of prior research linking earlier initiation of substance use with increased substance use severity, present findings suggest the importance of ACEs as individual-level determinants of increased opioid use severity. Efforts to prevent onset and escalation of opioid use among at-risk youth may benefit from trauma prevention programs and trauma-focused screening and treatment, as well as increased attention to ameliorating upstream socio-structural drivers of childhood trauma.

## Introduction

The epidemic of opioid misuse and associated health consequences, including opioid dependence, overdose, injection drug use, and hepatitis C infection, continues to be a major public health problem in the U.S. Young people are a population of particular concern in this ongoing epidemic. Opioid misuse, like other forms of substance use, is typically initiated in adolescence or young adulthood and often begins with the use of prescription opioids (POs) for non-medical reasons. For members of the Millennial generation in particular, non-medical PO use has played a prominent role in early drug initiation pathways. A recent analysis of 2013–2014 data from the National Survey on Drug Use and Health found that the lifetime prevalence of non-medical PO use was significantly higher - at 21.5% - among Millennials than among members of Generation X or Baby Boomers (Wall et al., 2018). While the prevalence and incidence of PO misuse in adolescents and young adults have declined somewhat in recent years after peaking in 2015 (Substance Abuse and Mental Health Services Administration [SAMHSA], 2020), these rates remain concerning, as do rates of opioid-associated morbidity and mortality in youth. In 2017, overdose fatalities in 15–24 year-olds reached an all-time high of 12.6 deaths per 100,000, with opioids involved in most of these overdoses (Hudgins et al., 2019).

Young people who engage in non-medical PO use are also more likely to use other drugs than youth who have not misused POs. In an analysis of National Survey on Drug Use and Health data from 2015–2016, adolescents and young adults with past-year PO misuse reported high rates of lifetime use of other drugs, including cocaine (35.5%), hallucinogens (49.4%), and inhalants (30.4%) (Hudgins et al., 2019). Research has also established a link between non-medical PO use and subsequent transition to heroin use and injection drug use (Lankenau et al., 2012; Cerda et al., 2015; Kolodny et al., 2015; Carlson et al., 2016; Surratt et al., 2017; Guarino et al., 2018), with up to 80% of new heroin users reporting PO misuse prior to initiating heroin use (Jones, 2013; Muhuri et al., 2013; Hudgins et al., 2019). Evidence indicates that transitions from non-medical PO use to heroin use have become increasingly common over the past two decades; national trend data show a temporal increase from 2002 to 2014 in the odds of heroin use among young people who used PO non-medically (Martins et al., 2017).

Moreover, emerging research suggests that the earlier in the life course PO misuse begins, the greater the risks may be. A national study by McCabe and colleagues found that onset of non-medical prescription drug use before age 14 was a significant predictor of later dependence on prescription drugs (McCabe et al., 2007). Other recent studies have shown that early initiation of non-medical PO use increases the likelihood of transition to heroin use in later adolescence or young adulthood (Cerda et al., 2015; Carlson et al., 2016).

Investigation into the multi-level factors that predispose some youth to initiate non-medical PO use at an early age, and to transition to more severe forms of drug use, has only recently begun. One plausible individual-level risk factor is childhood trauma, as it is well-documented that traumatic stress in childhood can have far-reaching adverse impacts on individuals' psycho-physiological development and well-being. Exposure to traumatic experiences in childhood, ranging from neglect, parental divorce and parental drug use to physical, emotional, and sexual abuse, has shown a strong, graded relationship with risk for a broad range of negative health outcomes in adulthood, from cancer and liver disease to depression, sexual risk behavior and use of illicit drugs (e.g., Felitti et al., 1998; Dube et al., 2006).

Despite a substantial body of research on the relationship between childhood trauma and substance use, only a few studies have examined potential linkages between adverse childhood experiences and the initiation of non-medical PO use or other forms of opioid use over the life course. For example, Merrick et al. (2020) found that experiencing three or more types of adverse experience in childhood significantly increased the risk of PO misuse in adulthood. In one notable study, Quinn et al. (2016) found a dose-response relationship between childhood trauma and PO misuse in which exposure to a greater number of trauma types was associated with progressively increased odds of initiating PO misuse in emerging or later adulthood.

Research exploring the relationship between childhood trauma and age at initiation of opioid or other drug use behaviors is especially limited. Dube et al. (2006) documented a persistent, graded relationship between extent of traumatic exposure in childhood and early initiation of illicit drug use, as well as lifetime risk of engaging in injection drug use, suggesting the additive contribution of multiple types of traumatic experience to the development of drug use problems from adolescence into adulthood (PO misuse was not examined). In another study, childhood sexual abuse among young adult drug injectors was independently associated with earlier age at first injection (Ompad et al., 2005). More recently, Stein et al. (2017)'s study of treatment-seeking persons with opioid use disorder found that exposure to more types of childhood trauma was inversely associated with age at opioid use initiation and positively associated with recent injection drug use and lifetime overdose; reflecting prior research, these were dose-response relationships indicating the cumulative impact of exposure to traumatic stress in early life.

Given the ongoing public health crisis of opioid use, overdose and associated health concerns, it is critically important to better understand the effect of childhood trauma on young people's vulnerability to non-medical PO use and related behaviors. The present study examines the association of number of different types of traumatic childhood experience with age of initiating a range of opioid use behaviors among young adult opioid users in New York City, the vast majority of whom began their opioid use trajectories with the non-medical use of POs. A more complete understanding of these associations may help inform the development and delivery of effective interventions for young people to prevent both the initiation of non-medical PO use and escalation to more severe forms of drug use.

## Materials and Methods

### Study Population and Procedures

This analysis used data collected from 539 participants recruited from July 2014 through April 2016 for a study of young people's opioid use patterns and trajectories and associated risk behaviors. Participants lived in one of the five boroughs of New York City, were aged 18–29, used POs non-medically and/or heroin in the past 30 days, spoke English, and provided written informed consent. Participants completed a computer-assisted, interviewer-administered interview lasting 90–120 min. Participants received $60 for completing the interview and additional incentives for each eligible participant they referred (see section Recruitment below). The Institutional Review Board of National Development and Research Institutes (NDRI) approved the study. Additional details on study procedures and sample description have been published elsewhere (Mateu-Gelabert et al., 2017; Guarino et al., 2018).

### Recruitment

Participants were recruited using Respondent-driven Sampling (RDS), a form of chain-referral sampling designed to engage hard-to-reach populations which uses personal social network connections to drive recruitment (Heckathorn, 1997; Heckathorn et al., 2002). A key feature of RDS is the generation of statistically principled estimates for a sample's target population based on sampling weights that correct for the unequal sampling probabilities inherent in sampling over a social network, as well as standard error estimation correcting for dependence induced by the sampling process (Heckathorn, 1997; Salganik and Heckathorn, 2004).

Using referrals from participants in our previous research (*n* = 4), other research studies (*n* = 9) and service providers (*n* = 3), as well as street recruitment (*n* = 4), a set of 20 eligible opioid users were directly recruited by research staff as RDS “seeds” to initiate recruitment chains. Per standard RDS protocol (WHO/UNAIDS, 2013), seeds were chosen to represent important subgroups of the target population known to researchers. After completing an eligibility screening and the structured interview, each seed was asked to refer to the study up to three eligible peers from their network of fellow opioid users. This peer-referral process was repeated with the seeds' recruits and for successive sampling waves thereafter. Of the 20 seeds, 12 referred eligible peers, thereby initiating recruitment chains. This analysis includes all 20 seeds within the sample of 539.

### Independent Variable: Adverse Childhood Experiences

Childhood trauma was assessed with the Adverse Childhood Experiences (ACE) Questionnaire, a 10-item instrument asking whether respondents experienced a broad range of traumatic experiences before the age of 18, including parental divorce, household dysfunction, and physical, emotional, and sexual abuse (see **Table 2** for a list of ACE items and their prevalence in this sample). While a total ACE score of 4 or higher has been found to robustly predict adverse physical and mental health consequences (Felitti et al., 1998; Centers for Disease Control Prevention, 2015), it was included in these analyses as an ordinal variable (range 0–10) to preserve potentially meaningful variation and allow us to investigate the relationship of cumulative trauma to the dependent variables. Additional analyses using a binary ACE independent variable (4 or more vs. 0–3) yielded an extremely similar pattern of results for all seven DVs in unadjusted and adjusted models.

### Dependent Variables: Age at Initiation of Opioid Use Behaviors

Seven dependent variables (DV) were explored, all related to age at initiation of opioid drug use and route of drug administration: non-medical use of POs; snorted POs; regular PO use; heroin use; regular heroin use; injected heroin; and injected POs (see **Table 3** for prevalence rates and ages at initiation). Regular use of POs and heroin was defined as one or more times per week for at least 1 month. Age at initiation of each DV was recoded as a binary variable (age in bottom 25th percentile vs. top 75th percentile [referent], that is, younger vs. older age at initiation).

### Sociodemographic Covariates

Age, gender, race, ethnicity, and household income during childhood were assessed as potential covariates by estimating their associations with each of the seven DVs in bivariable analyses (results not shown). Only age and gender were significantly associated with DVs (age with seven DVs and gender with three). All multivariable models were adjusted for age (continuous years) and gender (male vs. female [referent]); four transgender respondents' data were coded as missing.

### Analytic Strategy

SAS 9.4 (SAS Institute, Cary, North Carolina) was used for univariable descriptive analyses exploring the independent, dependent and socioeconomic variables and for bivariable analyses to identify covariates associated with the DVs. Logistic regression models were run in SAS to get estimated unadjusted (OR) and adjusted (AOR) odds ratios and 95% confidence intervals for the associations between number of ACEs and ages at initiation of the seven opioid use behaviors. Analyses included only those who initiated the behavior before their participation in the study; non-initiates as of the date of interview were excluded from regression models. The strength of each association was assessed by the magnitude of the OR/AOR and the width of the confidence interval.

RDS population estimates were produced in R version 3.2.2 (R Core Team, 2015). As a prerequisite for the calculation of population estimates, multiple imputation was conducted using the R package MICE (Van Buuren and Groothuis-Oudshoom, 2011) to impute missing network size data for a portion of the sample. Target population estimates for key variables were then calculated using the successive sampling estimator (Gile, 2011) in the R package RDS (Handcock et al., 2012), using a working population size of 15,000. Standard statistical tests violate assumptions in the RDS setting because respondents are recruited by other respondents, and pairs or clusters are more or less likely to be similar based on their relative positions in the sampling structure. However, in other analyses using this dataset, permutation tests yielded results very similar to standard results (Mateu-Gelabert et al., 2017), alleviating concerns about interdependence impacting estimates. Permutation testing and other methods for analyzing associations in RDS data are in their infancy and do not allow flexibility in how variables are coded or the ability to adjust for covariates in multivariable models. A large body of research findings from studies using an RDS design have used standard analytical tests and presented findings with the caveat that independence assumptions may not hold, as we do here.

## Results

### Participant Characteristics

Participants were predominantly male (68%), White (69%) and non-Hispanic/Latino (71%), with a mean age of 24.5 years. They represented diverse socioeconomic backgrounds, with 42% reporting an annual household income while growing up of $50,000 or under, 33% reporting $51,000–100,000, and 19% reporting more than $100,000. Full sample demographics, along with RDS-based estimates for the prevalence of these characteristics in the target population of 18–29 year-old opioid users in New York City, are presented in Table 1.

**Table 1:** Socio-demographic characteristics of young adult opioid users in New York City, 2014–16, *N* = 539.

**Characteristic**	**Sample prevalence** ** Percent (%)**	**Population estimate** ** % (± standard error)**
**Gender**		
Male	67.7	69.7 (4.1)
Female	31.5	29.7 (4.1)
Transgender	0.7	0.5 (0.3)
**Ethnicity**		
Hispanic/Latino	28.7	29.6 (4.3)
**Race**		
White	68.8	67.7 (4.5)
Black/African-American	7.8	9.1 (3.3)
Multiracial	8.0	6.1 (1.6)
American Indian or Alaskan Native	1.7	1.5 (0.9)
Asian	1.3	1.5 (0.7)
Did not respond^a^	12.4	14.3 (3.9)
**Household income growing up (annual)**		
< $50,000	42.1	43.5 (4.7)
$51,000–100,000	32.7	33.0 (4.0)
$101,000 or more	18.9	16.5 (2.7)
Did not respond	6.3	7.1 (2.30)
Age (years) *M (SD)*	24.5 (3.1) NA^b^	

a *Missing race data due to Hispanic/Latino respondents reporting ethnicity but not race*.

b *RDS package yields only frequency estimates*.

### Adverse Childhood Experiences

Eighty-nine percent of participants reported at least one adverse childhood experience (Table 2). Strikingly, 46% reported four or more adverse experiences (and the mean number of ACEs was 3.6 [SD = 2.6]), putting a large proportion at elevated risk for negative physical and psychological health consequences (Felitti et al., 1998). Prevalence was high among all ACEs, ranging from a low of 17% who reported that a parent or adult swore at or humiliated them or made them afraid of being physically hurt to 61% who had a depressed or mentally ill household member.

**Table 2:** Prevalence of adverse childhood experiences (ACE) among young adult opioid users in New York City, 2014–16, *N* = 539.

	**Sample prevalence** ***N* (%)**
**Type of ACE reported**	
Household member depressed/mentally ill	330 (61.2)
Household member went to prison	283 (52.5)
Parents separated/divorced	243 (45.1)
Felt as if no one in the family loved or supported them	229 (42.5)
Lived with someone who had a drinking/drug problem	188 (34.9)
Often felt they did not have enough to eat, were not protected	183 (34.0)
Mother/stepmother physically abused	139 (25.8)
Parent/adult fondled or touched them in a sexual way or had intercourse with them	128 (23.7)
Parent/adult pushed, slapped or threw something at them	106 (19.7)
Parent/adult swore at, humiliated or made them afraid of being physically hurt	89 (16.5)
**Total number of ACE types reported (categorical ACE)**	
0	58 (10.8)
1–3	234 (43.4)
4–6	159 (29.5)
7–10	88 (16.3)
**Total number of ACE types reported (ordinal ACE)**	**Mean (SD) Minimum-maximum**
	3.6 (2.6) 0–10

### Age at Initiation of Opioid Use Behaviors

Nearly all participants had used POs non-medically (only 8 were eligible for the study given their heroin, rather than PO, use), and among those, most initiated non-medical PO use in their teens (83%, not shown in tables), at an average of 16.9 years (Table 3). Most progressed to regular PO use (86%) and to snorting POs (73%). The prevalence of PO injection was the lowest (37%) among all DVs. Most participants initiated heroin use (82%) and subsequently reported regular heroin use (79%), and 64% reported heroin injection in their lifetime. The average ages at which participants reported initiating opioid behaviors increased in parallel with the severity of the behavior, from a mean age of 16.9 years at first non-medical PO use to first heroin injection at 20.4 years and first PO injection at 20.6 years.

**Table 3:** Descriptive statistics for age at initiation of opioid use behaviors among young adult opioid users in New York City, 2014–16.

		**Age at first use among initiates (years)**		
**Opioid use behavior^**a**^**	**Prevalence among total (*n* = 539) *N* (%)**	**Mean (SD)**	**Minimum-maximum**	**25th quartile**
Non-medical PO use	531 (98.5)	16.9 (3.1)	9–27	15
Snorted PO	394 (73.1)	17.9 (3.1)	10–28	16
Regular PO use	465 (86.3)	18.3 (3.1)	11–28	16
Heroin use	444 (82.3)	19.7 (3.5)	11–29	17
Regular heroin use	423 (78.5)	20.3 (3.4)	9–29	18
Injected heroin	345 (64.0)	20.4 (3.7)	11–29	18
Injected PO	197 (36.6)	20.6 (3.6)	13–28	18

a *PO, prescription opioid*.

### Relationships Between Number of ACEs and Age at Initiation of Opioid Use Behaviors

In unadjusted models, ACE number was significantly associated with younger age at initiation of opioid use behavior for all seven DVs. The increase in odds associated with every increase of one trauma ranged from 15 to 22% (Table 4). This pattern and the strength of the associations was very similar in multivariable models adjusted for age and gender. ACE number was significantly associated with six DVs, and the increased odds ranged from 13 to 23%. Only the association for PO injection was not statistically significant; the inclusion of the covariates as well as the small sample size due to the relatively low prevalence of this behavior (*n* = 196, 37%) likely affected the ability to observe a non-null association.

**Table 4:** Associations of number of adverse childhood events (ACE)^a^ and age at initiation of opioid use behaviors among young adult opioid users in New York City, 2014–16.

**Opioid use behavior^**b**^**	**Odds ratios for younger age at drug initiation** ***Referent=age in top 75th percentile***	
	**OR (95% CI)^**c**^**	**AOR (95% CI)^**d**^**
Non-medical PO use	1.22 (1.12, 1.32)	1.23 (1.12, 1.43)
Snorted PO	1.15 (1.05, 1.26)	1.16 (1.05, 1.28)
Regular PO use	1.20 (1.09, 1.32)	1.22 (1.10, 1.36)
Heroin use	1.20 (1.07, 1.43)	1.17 (1.03, 1.32)
Regular heroin use	1.15 (1.05, 1.26)	1.14 (1.03, 1.25)
Injected heroin	1.15 (1.05, 1.27)	1.13 (1.02, 1.25)
Injected PO	1.18 (1.03, 1.35)	1.12 (0.97, 1.30)

a *ACE modeled as ordinal variable with range 0–10*.

b *PO, prescription opioid*.

c *OR, odds ratio; CI, confidence interval; estimates represent the increase in odds of initiating drug use at younger age (bottom 25th percentile) for every 1-unit increase in ACE number*.

d *AOR, adjusted odds ratio; multivariable models include gender and age*.

## Discussion

In this large, RDS-based study of opioid-using young adults, adverse childhood experiences were highly prevalent, and number of traumatic exposure types was significantly associated with early onset of a broad range of opioid use behaviors, providing further support for an additive effect of exposure to different types of trauma on the initiation and development of youth's substance use trajectories. Earlier substance use initiation has been linked in multiple studies to the development of greater-severity drug use problems and progression to riskier forms of use, such as heroin use and drug injection (McCabe et al., 2007; Grella and Lovinger, 2011; Cerda et al., 2015; Carlson et al., 2016). This knowledge, coupled with our findings and other emerging evidence of trauma's association with opioid misuse, highlights the need to incorporate prevention and early detection of trauma into drug use prevention and treatment programs.

The prevalence of adverse childhood experiences among young adults in this study, with 89% of participants reporting at least one traumatic experience and nearly half reporting 4 or more types of trauma, is markedly higher than for general population samples (64% with 1 ACE and 12% with 4 or more; Centers for Disease Control Prevention, Kaiser Permanente, 2016) and many other substance-using populations, but roughly comparable to rates reported for other opioid-dependent groups. A study of the trauma profiles of non-treatment-seeking, substance-dependent adults found a much higher prevalence of childhood trauma in the PO-dependent group (90%) than in the cocaine-dependent group (60%; Lawson et al., 2013). In another study, 80% of opioid-dependent outpatients seeking buprenorphine treatment reported any experience of childhood trauma (Sansone et al., 2009). Similarly, this sample's mean ACE score (3.6, SD = 2.6) is remarkably close to that reported in a recent study of treatment-seeking persons with opioid use disorder (3.64, SD = 2.75; Stein et al., 2017). Taken together, these findings suggest that opioids may be particularly appealing as a drug of choice for individuals with a history of trauma as they may exert a similar palliative effect on psychological pain as they do on physical pain (Rosenblum et al., 2008). There is some compelling qualitative evidence to support this; Scottish drug injectors reported that “heroin injection was an effective means of blotting out distressing thoughts and feelings” related to early trauma (Hammersley et al., 2016).

Of particular note are the findings demonstrating a consistent, gradient pattern of association in which each one-unit increase in ACE score is associated with increased odds (ranging from 12 to 23%) of early initiation across opioid use behaviors. These findings support and extend the results of previous research that has found childhood trauma to have strong, dose-dependent relationships with early initiation of illicit drug use, including opioid misuse, and the likelihood of engaging in injection drug use and experiencing non-fatal overdose (Dube et al., 2006; Felitti and Anda, 2009; Stein et al., 2017), as well as with younger age at first injection (Ompad et al., 2005).

The present study contributes to emerging research on childhood trauma and opioid misuse by focusing on the new generation of young opioid users who were introduced to opioids via the non-medical use of POs. The predominance of males and Whites in the sample is consistent with the demographic patterns of PO misuse among U.S. young adults in 2014–2016, when these data were collected (Hudgins et al., 2019). However, more recent national data suggest that these demographic patterns may be shifting. For example, in 2019, more female than male, and more Black and Hispanic than White, high school students reported both current and lifetime PO misuse (Jones et al., 2020).

The study also advances existing research by documenting associations of childhood trauma with earlier initiation of a series of interrelated forms of opioid use, from first experience of non-medical PO use, first intranasal PO use, and initiation of regular PO misuse to first heroin use, onset of regular heroin use, first heroin injection, and first PO injection. Knowledge of the substantial co-occurrence among these opioid use behaviors, indeed a trajectory of behaviors that put individuals at progressively greater risk of not only opioid use disorder and overdose, but also HCV, HIV and bacterial infections (for those who progress to injection), is valuable information that may help optimize the content and timing of prevention, treatment and harm reduction programming for young populations. Other analyses of this dataset have shown that PO injection is associated with elevated risk for overdose and HCV infection relative to heroin injection (Mateu-Gelabert et al., 2020), further supporting the concept of a trajectory of opioid use behaviors characterized by escalating severity and increasing health risks.

A potential mechanism underlying the observed associations between adverse childhood experiences and early non-medical PO use and other opioid use behaviors, is self-medication, in which individuals use psychoactive substances as a form of avoidant coping, to alleviate distressing emotional states resulting from traumatic experience (Khantzian, 1997; Khantzian and Albanese, 2008). Given the established associations between early drug use initiation and greater problem severity, earlier onset of PO misuse may in turn increase the likelihood of earlier progression to greater-intensity use and earlier transition to heroin use and drug injection, effectively priming youth for an accelerated trajectory of opioid and, frequently, poly-substance use. Additionally, a wide-ranging body of research has shown that exposure to traumatic stress early in life can interfere with typical developmental processes, potentially leading to neurocognitive, psychological and social impairments (Weiss and Wagner, 1998; U.S. Department of Health Human Services, 2001). Thus, the negative impacts of early opioid misuse on youth development may compound the deleterious effects of early traumatic exposure. Clarifying the behavioral and psychosocial pathways from adversity to drug use initiation is a crucial next step for research in this area, as is investigation into their interactive effects. Future research should also examine the impact of specific types of adverse childhood experiences on the likelihood of early onset and age at initiation of opioid misuse behaviors.

### Limitations

Given the cross-sectional nature of this study, findings only establish correlation, not causation. Therefore, caution is warranted in attributing differences in age of initiation to childhood trauma; however, the consistency of present results with prior research does support this interpretation. Generalizability of findings is limited by the inclusion in the sample of residents of New York City only, who likely differ socio-demographically and perhaps in terms of trauma exposure and drug use patterns from other U.S. subpopulations. Nonetheless, understanding this urban population's risk factors and drug use trajectory is critical given the city's size and ongoing opioid epidemic. Bias may also have been introduced by the use of RDS as a recruitment methodology, due to the dependence in the sample (as participants are recruited by other participants), as well as by the non-random selection of participants to serve as RDS seeds. Another limitation concerns the nature of self-report data, which may be vulnerable to recall and social desirability bias. We were not able to investigate the contribution of age of traumatic experience to early initiation because of challenges in linking traumatic experiences to specific dates. Indeed, recall bias that stems from asking adults about childhood experiences and difficulties in asking young children about adversity pose challenges for clarifying the influence of age of trauma on subsequent outcomes, but this is an important next step that is needed to clarify pathways and inform prevention and treatment strategies. Finally, although the goal of this analysis was to investigate the combined role of a broad range of traumatic events, the relationship of particular events to opioid initiation are also worthy of exploration.

### Public Health Implications

These findings underscore the importance of prevention, early detection and treatment of both childhood trauma and opioid misuse among youth. Pediatricians, adolescent medicine specialists and others who work with youth should be made aware of the close links between traumatic exposure and early-onset use of POs and other drugs (both pharmaceutical and illicit), so they can incorporate screening for both into their practice as appropriate. Focused efforts to address trauma-related issues early in the life course may serve as a means to prevent or delay the uptake of opioid and other substance use. Interventions for groups vulnerable to adversity could promote resilience as a way of buffering youth from the far-reaching negative impacts of traumatic stress. The high prevalence of adverse childhood experiences documented among opioid-using young adults in this and other research suggests that routine screening for early traumatic exposure among young people seeking treatment for opioid use disorder may be warranted, so that integrated – and potentially more effective – mental health and substance use treatment can be delivered. Results further suggest the potential value of developing and implementing trauma-informed behavioral interventions for opioid-using adolescents and young adults. Training youth in adaptive coping skills to better manage the psychosocial and emotional repercussions of traumatic stress may enhance efforts to prevent escalation of use, transition to heroin and/or injection drug use and exposure to HIV and HCV. However, such individual-level interventions should not substitute for the larger project of ameliorating the upstream socio-structural conditions that help perpetuate childhood trauma and distribute its harms inequitably, often exacting the greatest toll on those most vulnerable.

## Data Availability Statement

The dataset presented in this article is not readily available due to the sensitive nature of the dataset (containing information about illegal drug use, sexual behavior, HIV and HCV status, etc.) and the potentially damaging consequences to study participants if their identities were to become known; therefore, the data cannot be shared outside the research team. Requests to access the dataset should be directed to Honoria Guarino, honoria.guarino@sph.cuny.edu.

## Ethics Statement

The study involving human participants was reviewed and approved by National Development and Research Institutes Institutional Review Board. The participants provided their written informed consent to participate in this study.

## Author Contributions

PM-G and SS conceptualized the general approach of this study and KR conducted initial analyses. KQ and HG revised the analytic plan and KQ conducted the final analyses reported here. CS, EG, and LJ collected the data on which the study is based. SS made significant contributions to initial literature reviews and an early version of the manuscript. HG wrote the current version of the manuscript, with assistance from KQ, particularly in the Methods and Materials and Results sections. SF provided critical feedback on an interim version of the manuscript. All authors have contributed to revising and have approved the manuscript.

## Conflict of Interest

The authors declare that the research was conducted in the absence of any commercial or financial relationships that could be construed as a potential conflict of interest.

## Abbreviations

ACE: adverse childhood experience
DV: dependent variable
HCV: hepatitis C virus
PO: prescription opioid
RDS: Respondent-Driven Sampling.

## References

[B1] Carlson R. G. Nahhas R. W. Martins S. S. Daniulaityte R. (2016). Predictors of transition to heroin use among initially non-opioid dependent illicit pharmaceutical opioid users: a natural history study. Drug Alcohol Depend. 160, 127–134. 10.1016/j.drugalcdep.2015.12.026 26785634 PMC4767561

[B2] Centers for Disease Control Prevention (2015). Behavioral Risk Factor Surveillance System ACE Module Data, 2010. Atlanta, GA: U.S. Department of Health and Human Services, Centers for Disease Control and Prevention. Available online at: http://www.cdc.gov/violenceprevention/acestudy (accessed May 26, 2019).

[B3] Centers for Disease Control Prevention Kaiser Permanente. (2016). The ACE Study Survey Data [Unpublished Data]. Atlanta, GA: U.S. Department of Health and Human Services, Centers for Disease Control and Prevention. Available online at: http://www.cdc.gov/violenceprevention/acestudy/about (accessed July 26, 2016).

[B4] Cerda M. Santaella J. Marshall B. D. L. Kim J. H. Martins S. S. (2015). Nonmedical prescription opioid use in childhood and early adolescence predicts transitions to heroin use in young adulthood: a national study. J. Pediatrics 167, 605–612. 10.1016/j.jpeds.2015.04.071 26054942 PMC4714948

[B5] Dube S. R. Miller J. W. Brown D. W. Giles W. H. Felitti V. J. Dong M.. (2006). Adverse childhood experiences and the association with ever using alcohol and initiating alcohol use during adolescence. J. Adolesc. Health 38, 1–10. 10.1016/j.jadohealth.2005.06.006 16549308

[B6] Felitti V. J. Anda R. (2009). The relationship of adverse childhood experiences to adult medical disease, psychiatric disorders, and sexual behavior: implications for healthcare, in The Hidden Epidemic: The Impact of Early Life Trauma on Health and Disease, eds Lanius R. Vermetten E. Pain C.. Available online at: http://www.acestudy.org/yahoo_site_admin/assets/docs/LaniusVermetten_FINAL_8-26-09.12892303.pdf (accessed May 26, 2019).

[B7] Felitti V. J. Anda R. F. Nordenberg D. Williamson D. F. Spitz A. K. Edwards V.. (1998). Relationship of childhood abuse and household dysfunction to many of the leading causes of death in adults. Am. J. Prevent. Med. 14, 245–258. 10.1016/S0749-3797(98)00017-8 9635069

[B8] Gile K. (2011). Improved inference for Respondent-Driven Sampling data with application to HIV prevalence estimation. J. Am. Stat. Assoc. 106, 135–146. 10.1198/jasa.2011.ap09475

[B9] Grella C. E. Lovinger K. (2011). 30-year trajectories of heroin and other drug use among men and women sampled from methadone treatment in California. Drug Alcohol Depend. 118, 251–258. 21549528 10.1016/j.drugalcdep.2011.04.004 PMC3156933

[B10] Guarino H. Mateu-Gelabert P. Teubl J. Goodbody E. (2018). Young adults' opioid use trajectories: from nonmedical prescription opioid use to heroin, drug injection, drug treatment and overdose. Addict. Behav. 86, 18–23. 10.1016/j.addbeh.2018.04.017 29747875 PMC6377245

[B11] Hammersley R. Dalgano P. McCollum S. Reid M. Strike Y. Smith A.. (2016). Trauma in childhood stories of people who have injected drugs. Addict. Res. Theory 24, 135–151. 10.3109/16066359.2015.1093120

[B12] Handcock M. S. Fellows I. E. Gile K. J. (2012). RDS: Respondent-Driven Sampling, version 0.7. Available online at: http://CRAN.R-project.org/package=RDS (accessed September 30, 2019).

[B13] Heckathorn D. D. (1997). Respondent-Driven Sampling: a new approach to the study of hidden populations. Social Prob. 44, 174–199. 10.2307/3096941

[B14] Heckathorn D. D. Semaan S. Broadhead R. S. Hughes J. J. (2002). Extensions of Respondent-Driven Sampling: a new approach to the study of injection drug users aged 18-25. AIDS Behav. 6, 55–67. 10.1023/A:1014528612685

[B15] Hudgins J. D. Porter J. J. Monuteaux M. C. Bourgeois F. T. (2019). Prescription opioid use and misuse among adolescents and young adults in the United States: a national survey study. PLoS Med. 16:e1002922. 10.1371/journal.pmed.1002922 31689290 PMC6830740

[B16] Jones C. M. (2013). Heroin use and heroin use risk behaviors among nonmedical users of prescription opioid pain relievers—United States, 2002-2004 and 2008-2010. Drug Alcohol Depend. 132, 95–100. 10.1016/j.drugalcdep.2013.01.007 23410617

[B17] Jones C. M. Clayton H. B. Deputy N. P. Roehler D. R. Ko J. Y. Esser M. B.. (2020). Prescription opioid misuse and use of alcohol and other substances among high school students – Youth Risk Behavior Survey, United States, 2019. MMWR 69, 38–46. 10.15585/mmwr.su6901a5 32817608 PMC7440199

[B18] Khantzian E. J. (1997). The self-medication hypothesis of substance use disorders: a reconsideration and recent applications. Harvard Rev. Psychiatry 4, 231–244. 10.3109/10673229709030550 9385000

[B19] Khantzian E. J. Albanese M. J. (2008). Understanding Addiction as Self-Medication: Finding Hope Behind the Pain. Maryland: Rowman and Littlefield Publishers.

[B20] Kolodny A. Courtwright D. T. Hwang C. S. Kreiner P. Eadie J. L. Clark T. W.. (2015). The prescription opioid and heroin crisis: a public health approach to an epidemic of addiction. Ann. Rev. Public Health 36, 559–574. 10.1146/annurev-publhealth-031914-122957 25581144

[B21] Lankenau S. E. Teti M. Silva K. Bloom J. J. Harocopos A. Treese M. (2012). Patterns of prescription drug misuse among young injection drug users. J. Urban Health 89, 1004–1016. 10.1007/s11524-012-9691-9 22684424 PMC3531346

[B22] Lawson K. M. Back S. E. Hartwell K. J. Moran-Santa M. Brady K. T. (2013). A comparison of trauma profiles among individuals with prescription opioid, nicotine or cocaine dependence. Am. J. Addict. 22, 127–131. 10.1111/j.1521-0391.2013.00319.x 23414497 PMC3681508

[B23] Martins S. S. Sarvet A. Santaella-Tenorio J. Saha T. Grant B. F. Hasin D. S. (2017). Changes in US lifetime heroin use and heroin use disorder: prevalence from the 2001-2002 to 2012-2013 National Epidemiologic Survey on Alcohol and Related Conditions. JAMA Psychiatry 74, 445–455. 10.1001/jamapsychiatry.2017.0113 28355458 PMC5470460

[B24] Mateu-Gelabert P. Guarino H. Zibbell J. Teubl J. Fong C. Goodbody E.. (2020). Prescription opioid injection among young people who inject drugs in New York City: a mixed-methods description and associations with hepatitis C infection and overdose. Harm Reduct. J. 17:22. 10.1186/s12954-020-00367-2 32228700 PMC7106794

[B25] Mateu-Gelabert P. Jessell L. Goodbody E. Kim D. Gile K. Teubl J.. (2017). High enhancer, downer, withdrawal helper: multifunctional nonmedical benzodiazepine use among young adult opioid users in New York City. Int. J. Drug Policy 46, 17–27. 10.1016/j.drugpo.2017.05.016 28577506 PMC5609816

[B26] McCabe S. E. West B. T. Morales M. Cranford J. A. Boyd C. J. (2007). Does early onset of non-medical use of prescription drugs predict subsequent prescription drug abuse and dependence? Results from a national study. Addiction 102, 1920–1930. 10.1111/j.1360-0443.2007.02015.x 17916222 PMC2377405

[B27] Merrick M. Ford D. Haegerich T. Simon T. (2020). Adverse childhood experiences increase risk for prescription opioid misuse. J. Primary Prevent. 41, 139–152. 10.1007/s10935-020-00578-0 31989435 PMC10976456

[B28] Muhuri P. K. Gfroerer J. C. Davies M. C. (2013). CBHSQ Data Review: Associations of Nonmedical Pain Reliever Use and Initiation of Heroin Use in the United States. Rockville, MD: Center for Behavioral Health Statistics and Quality, SAMHSA.

[B29] Ompad D. C. Ikeda R. M. Shah N. Fuller C. M. Balley S. Morse E.. (2005). Childhood sexual abuse and age at initiation of injection drug use. Am. J. Public Health 95, 703–709. 10.2105/AJPH.2003.019372 15798133 PMC1449244

[B30] Quinn K. Boone L. Scheidell J. D. Mateu-Gelabert P. McGorroy S. P. Beharie N.. (2016). The relationships of childhood trauma and adulthood prescription pain reliever misuse and injection drug use. Drug Alcohol Depend. 169, 190–198. 10.1016/j.drugalcdep.2016.09.021 27816251 PMC5728665

[B31] R Core Team (2015). R: a language and environment for statistical computing. Vienna, Austria. Available online at: https://www.R-project.org (accessed September 30, 2019).

[B32] Rosenblum A. Marsch L. A. Joseph H. Portenoy R. K. (2008). Opioids and the treatment of chronic pain: controversies, current status, and future directions. Exp. Clin. Psychopharmacol. 16, 401–416. 10.1037/a0013628 18837637 PMC2711509

[B33] Salganik M. J. Heckathorn D. D. (2004). Sampling and estimation in hidden populations using Respondent-Driven Sampling. Sociol. Methodol. 34, 193–239. 10.1111/j.0081-1750.2004.00152.x

[B34] Sansone R. A. Whitecar P. Widerman M. W. (2009). The prevalence of childhood trauma among those seeking buprenorphine treatment. J. Addict. Dis. 28, 64–67. 10.1080/10550880802545101 19197597

[B35] Stein M. Conti M. Kenney S. Anderson B. Flori J. Risi M.. (2017). Adverse childhood experience effects on opioid use initiation, injection drug use, and overdose among persons with opioid use disorder. Drug Alcohol Depend. 179, 325–329. 10.1016/j.drugalcdep.2017.07.007 28841495 PMC5599365

[B36] Substance Abuse Mental Health Services Administration [SAMHSA] (2020). Key substance use and mental health indicators in the United States: results from the 2019 National Survey on Drug Use and Health (HHS Publication No. PEP20-07- 01-001, NSDUH Series H-55). Rockville, MD: Center for Behavioral Health Statistics and Quality, Substance Abuse and Mental Health Services Administration. Available online at: https://www.samhsa.gov/data/ (accessed October 22, 2020).

[B37] Surratt H. L. Kurtz S. P. Buttram M. Levi-Minzi M. A. Pagano M. E. Cicero T. J. (2017). Heroin use onset among nonmedical prescription opioid users in the club scene. Drug Alcohol Depend. 179, 131–138. 10.1016/j.drugalcdep.2017.06.034 28772173 PMC5599357

[B38] U.S. Department of Health Human Services Administration on Children Youth, Families. (2001). Understanding the Effects of Maltreatment on Early Brain Development. Washington DC: Government Printing Office. Available online at: http://www.childwelfare.gov/pubs/focus/earlybrain/earlybrain.pdf (accessed May 26, 2019).

[B39] Van Buuren S. Groothuis-Oudshoom K. (2011). MICE: multivariate imputation by chained equations in R. J. Stat. Softw. 45, 1–67. 10.18637/jss.v045.i03

[B40] Wall M. Cheslack-Postava K. Hu M.-C. Feng T. Griesler P. Kandel D. B. (2018). Nonmedical prescription opioids and pathways of drug involvement in the US: generational differences. Drug Alcohol Depend. 182, 103–111. 10.1016/j.drugalcdep.2017.10.013 29220668 PMC5870126

[B41] Weiss M. J. Wagner S. H. (1998). What explains the negative consequences of adverse childhood experiences on adult health? Insights from cognitive and neuroscience research. Am. J. Prev. Med. 14, 356–360. 10.1016/S0749-3797(98)00011-7 9635084

[B42] WHO/UNAIDS (2013). Introduction to HIV/AIDS and sexually transmitted infection surveillance: Module 4: Introduction to Respondent-Driven Sampling. Geneva, Switzerland. Available online at: http://applications.emro.who.int/dsaf/EMRPUB_2013_EN_1539.pdf (accessed August 22, 2019).

